# Bumetanide Enhances Phenobarbital Efficacy in a Rat Model of Hypoxic Neonatal Seizures

**DOI:** 10.1371/journal.pone.0057148

**Published:** 2013-03-11

**Authors:** Ryan T. Cleary, Hongyu Sun, Thanhthao Huynh, Simon M. Manning, Yijun Li, Alexander Rotenberg, Delia M. Talos, Kristopher T. Kahle, Michele Jackson, Sanjay N. Rakhade, Gerard Berry, Frances E. Jensen

**Affiliations:** 1 Department of Neurology, Children’s Hospital Boston, Boston, Massachusetts, United States of America; 2 Division of Genetics and Metabolism, Children’s Hospital Boston, Boston, Massachusetts, United States of America; 3 Division of Newborn Medicine, Brigham and Women’s Hospital, Boston, Massachusetts, United States of America; 4 Program in Neurobiology, Harvard Medical School, Boston, Massachusetts, United States of America; 5 Department of Neurosurgery, Massachusetts General Hospital and Harvard Medical School, Boston, Massachusetts, United States of America; McGill University, Canada

## Abstract

Neonatal seizures can be refractory to conventional anticonvulsants, and this may in part be due to a developmental increase in expression of the neuronal Na^+^-K^+^-2 Cl^−^ cotransporter, NKCC1, and consequent paradoxical excitatory actions of GABA_A_ receptors in the perinatal period. The most common cause of neonatal seizures is hypoxic encephalopathy, and here we show in an established model of neonatal hypoxia-induced seizures that the NKCC1 inhibitor, bumetanide, in combination with phenobarbital is significantly more effective than phenobarbital alone. A sensitive mass spectrometry assay revealed that bumetanide concentrations in serum and brain were dose-dependent, and the expression of NKCC1 protein transiently increased in cortex and hippocampus after hypoxic seizures. Importantly, the low doses of phenobarbital and bumetanide used in the study did not increase constitutive apoptosis, alone or in combination. Perforated patch clamp recordings from *ex vivo* hippocampal slices removed following seizures revealed that phenobarbital and bumetanide largely reversed seizure-induced changes in E_GABA._ Taken together, these data provide preclinical support for clinical trial**s** of bumetanide in human neonates at risk for hypoxic encephalopathy and seizures.

## Introduction

Neonatal seizures occur most commonly in the setting of perinatal asphyxia and hypoxic-ischemic encephalopathy (HIE), and can be resistant to conventional antiepileptic therapies. Refractory neonatal seizures increase risk of subsequent epilepsy and neurocognitive morbidity. [1] As there are often few behavioral manifestations of neonatal seizures, electroencephalographic (EEG) monitoring is required for diagnosis due to the occurrence of “electroclinical dissociation”. [2]–[4] Phenobarbital and phenytoin remains the mainstay of therapy, although there is little evidence that these agents significantly suppress ongoing seizure activity or change long-term outcome. The lack of response to conventional antiepileptic drugs (AEDs) that are otherwise effective in older children and adults is at least in part due to maturational differences in factors regulating neuronal network excitability. [5], [6].

The neonatal period is one of heightened synaptic plasticity and synaptogenesis during brain development. Excitatory ionotropic glutamate receptors are expressed at higher levels than in later life, while the expression of classical inhibitory γ-amino-butyric acid (GABA) receptors is significantly lower than adult. [5], [7], [8] In normal adult brain, activation of GABA_A_ receptors results in membrane hyperpolarization due to Cl^−^ influx through its ion channel, and hence are inhibitory. [9] In immature neurons, however, GABA agonists can cause depolarization due to a net efflux of Cl^−^ through the GABA receptor ion channel, resulting in neuronal excitation. [10], [11] This switch is thought to be in part due to developmental changes in the expression of two proteins involved in the maintenance of intracellular Cl^−^ homeostasis in neurons: the Na^+^-K^+^-2 Cl^−^ cotransporter isoform 1 (NKCC1) that transports Cl^−^ into the cell, and the K^+^-Cl^−^ cotransporter isoform 2 (KCC2) that moves Cl^−^ out of the cell. [12] Importantly, reversal of GABA_A_ receptor polarity appears much later in male than in female rats, [13], [14] but in order to maintain continuity with our previous studies [15]–[17] we focus on male rats. In the immature brain, neuronal intracellular Cl^−^ concentrations are higher than in the adult due to a high NKCC1 expression coincident with a low KCC2 expression, relative to normal adult expression patterns. [5], [17] The expression of NKCC1 mRNA is also increased in human forebrain neurons during the perinatal period, relative to later life. [17], [18] In humans, this switch is thought to occur in utero after NKCC1 peaks between 31–41 postconceptional weeks, whereas in rats this switch occurs near the end of the second postnatal week, with NKCC1 expression decreasing after postnatal day (P)14. [17] Other studies have confirmed that the functional correlate of this switch, the appearance of hyperpolarizing GABA_A_ receptors, also occurs around P14. [13], [14] Additionally, the caudal to rostral maturation of these transporters [4], [17] is thought to contribute to the electroclinical dissociation seen in neonates after treatment with phenobarbital.

NKCC1 potentially represents an age-specific therapeutic target and is postulated to contribute to the relative lack of efficacy of GABA_A_ receptor agonists in newborns. [19] Bumetanide is an inhibitor of both NKCC isoforms (1 and 2), and is FDA approved and clinically in use as a diuretic in all age groups, including neonates, [20], [21] as NKCC2 is expressed in the renal tubule cells in the loop of Henle. However, NKCC2 is not expressed in the brain and hence bumetanide actions in neurons depend on the presence of NKCC1, which is broadly expressed throughout the body, including in neurons. [11] Bumetanide is currently under study in a Phase I clinical trial as a single add-on therapy in neonatal seizures in HIE infants 33–44 weeks of age (clinicaltrials.gov/NCT00830531). To further support potential translation, we performed an evaluation of the efficacy of phenobarbital alone versus in combination with bumetanide in an established neonatal rat model of hypoxic encephalopathy and seizures using behavioral and EEG outcomes. [15], [22] To better align with the clinical trial, we measured serum and brain levels of bumetanide at seizure suppressing doses using a sensitive stable isotope dilution liquid chromatography/tandem mass spectrometric (LC-MS/MS) method. [23] We examined whether seizures acutely altered NKCC1 and KCC2 protein expression in cortex and hippocampus, or GABA_A_ receptor function in *ex vivo* hippocampal slices following hypoxia-induced seizures (HS) in immature rats. Finally, as some AEDs induce apoptosis in developing cortex, [24] we evaluated apoptosis after seizure-suppressing doses of phenobarbital and bumetanide.

## Methods

### Ethics Statement

All procedures were in accordance with the guidelines of the National Institutes of Health’s *Guide for the Care and Use of Laboratory Animals*. The protocol was approved by the Animal Care and Use Committee at Children’s Hospital Boston (protocol number: 11-05-1952R). We made all efforts to minimize animal suffering and the number of animals used.

### Animals

Male Long Evans rat pups (Charles River Laboratories, MA) were maintained in a temperature-controlled animal care facility with a 12 hr light-dark cycle.

### Hypoxia-induced Seizures and Drug Administration

P10 rat pups were exposed to nonlethal graded global hypoxia for 15 min in an airtight chamber as previously described. [15], [22] Briefly, oxygen concentration was maintained at 7% for 8 min, 5% for 6 min, and 4% for 1 min before termination of hypoxia. Each animal was scored for the number of tonic-clonic seizures during the 15 min hypoxic episode. Littermate controls were separated from their dams and kept at room air for the duration of the experiment. Body temperature was maintained between 32–34°C, and all rats were returned to their dams within two hr.

Prior to seizure induction, phenobarbital (15 mg/kg) and/or bumetanide 0.15 mg/kg (low dose) or 0.3 mg/kg (high dose) or vehicle were administered to P10 rat pups (18–22 g) by intraperitoneal (i.p.) injection. Phenobarbital was diluted in 0.9% saline, and bumetanide (MP Biomedical) was dissolved in DMSO, then diluted in 0.9% saline. Phenobarbital and bumetanide were administered as separate injections at intervals 30 and 15 min prior to hypoxia, respectively. Five treatment groups and one vehicle group were tested: phenobarbital alone, bumetanide alone (low or high dose), and phenobarbital in combination with either of the two bumetanide doses. Rats in the phenobarbital only and bumetanide only groups received an additional vehicle i.p. injection (DMSO in 0.9% NaCl or 0.9% NaCl) of the same volume they would have received if they were in a combined treatment group. Vehicle-treated rats received two vehicle i.p. injections (DMSO in 9% NaCl and 0.9% NaCl) of the same volume as those in the combined treatment group.

### Behavioral and Electrographic Assessment of Seizures during Hypoxia

**Figure 1:**
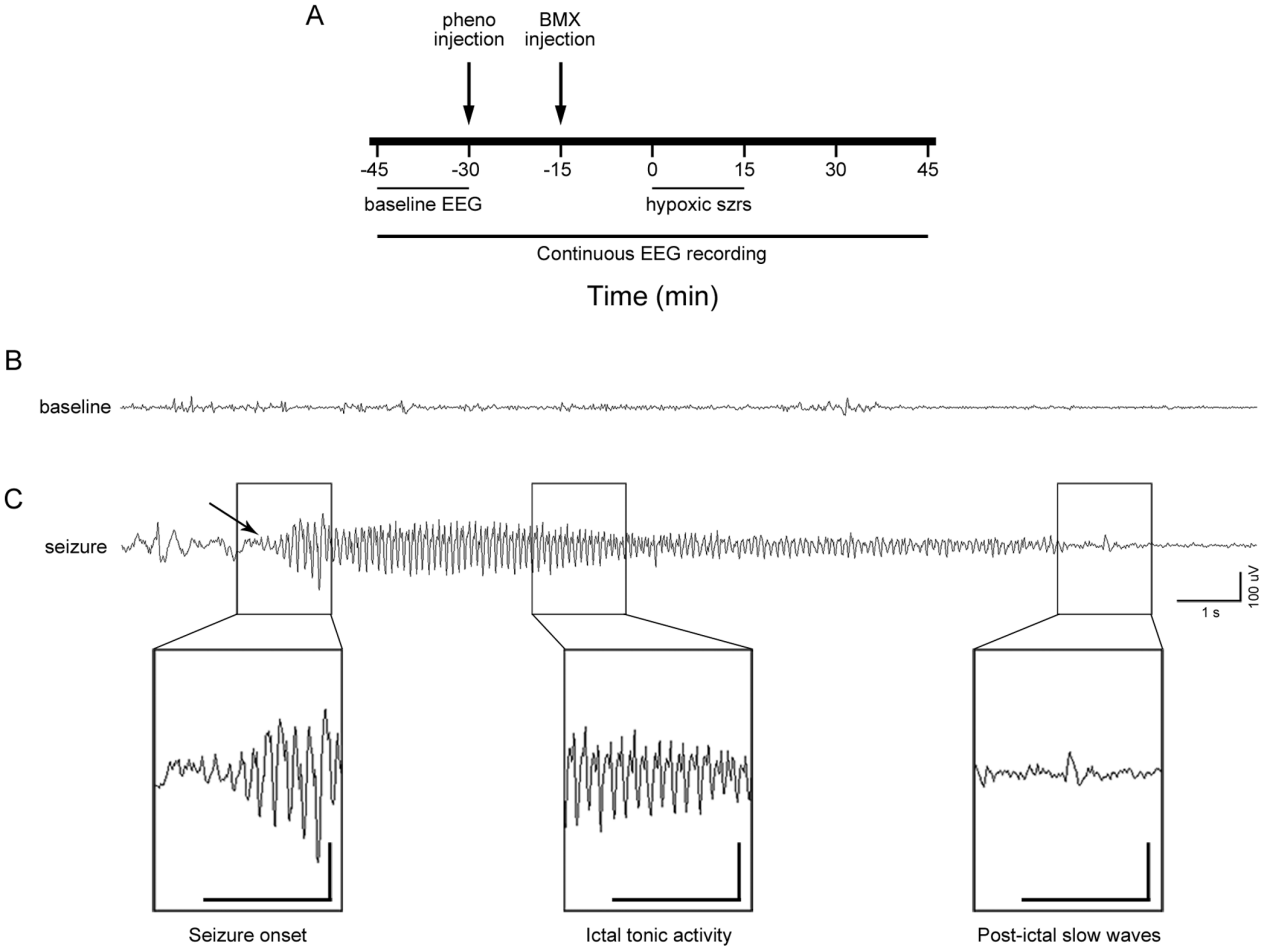
EEG recording schedule and representative electrographic seizure. (A) After SWE implantation, continuous video-EEG was recorded from each rat for a total of 90 min. Recordings began 45 min before the start of hypoxia and ran an additional 30 min after the termination of hypoxia. Phenobarbital (pheno) was injected 30 min before hypoxia, and was followed by bumetanide (BMX) injection 15 min later. Epochs totaling 5 min were selected from the recording during hypoxia for EEG power analysis. Random selections from the baseline recording were used as controls. (B) Representative baseline EEG recording from a P10 rat prior to treatment and exposure to graded global hypoxia. (C) Typical electrographic seizure recorded from one hemisphere (other hemisphere not shown) in a P10 rat during the course of hypoxia. All electrographic seizures were accompanied by behavioral automatisms. Arrow indicates seizure onset. Insets of the EEG trace in an expanded time scale show seizure initiation, followed by a progressive buildup in spikes of increasing frequency and amplitude, regular tonic spike wave discharges, and post-ictal slowing following seizure termination.

Seizures during hypoxia were videotaped and reviewed by an investigator blind to treatment, and scored for the number and cumulative duration of tonic-clonic seizures during hypoxia. A subgroup of rats was monitored by video-electroencephalogram (video-EEG) before, during, and after hypoxia. Continuous video-EEG was acquired using three thin silver/silver-chloride Teflon coated subcutaneous wire electrodes (SWE) for a total of 90 min for each rat (Fig. 1A). [15], [25] A reference contact was positioned over the dorsal snout at midline, with two active contacts in the scalp over the parietal regions bilaterally. A fourth electrode was placed in the skin of the torso to record EKG, and a ground electrode was placed in the lower back. SWE implantation causes only minimal, momentary pain, and is well tolerated by the rat pups. [15] Video-EEG monitoring was performed in a Plexiglas recording chamber, and animals were allowed free movement around the recording chamber.

Analysis of EEG was performed on amplified (x10,000) signals digitized at 200 Hz, filtered using a digital 32-channel EEG-recording apparatus from Stellate Systems Inc. (Natus Medical Systems, CA), and displayed in a referential montage for post hoc analysis by visual inspection. An investigator blind to treatment conditions reviewed EEG recordings to assess seizure activity. Seizures were defined as electrographic seizures recorded from both hemispheres, only when they consisted of paroxysmal rhythmic spikes of high amplitude, diffuse frequency of ≥ 8 Hz lasting ≥ 3 s (Figure 1), and were accompanied by a behavioral correlate, such as head bobbing, wet dog shakes, and tonic-clonic movements, similar to previous report. [15] EEG power analysis was performed using Persyst software (Persyst Inc. Tucson, AZ) on 5 min epochs during the baseline pre-hypoxia and then during hypoxia exposure (see Methods S1). Time frequency analysis of EEG power was carried out by consecutive 10-second Fast-Fourier transform (FFT) analysis. EEG was filtered using 1 Hz high-pass and 60 Hz low-pass filtering to remove ambient noise, binned in 20 s intervals, and power within the epoch was determined. The summed power was then calculated for frequency bands 4–12 Hz, and 12–32 Hz.

### Assessment of Serum and Brain Bumetanide Concentrations

Control rats were treated with phenobarbital and bumetanide (0.15 or 0.3 mg/kg), anesthetized (100 mg/kg pentobarbital), and perfused transcardially with 0.9% saline at 10, 30, 60, and 120 min following bumetanide injection. An additional group of rats was also exposed to global hypoxia prior to sacrifice, using the treatment paradigm described earlier. The 10 min group could only be collected for control rats due to the treatment paradigm. Blood was collected by cardiac puncture before perfusion, then clotted to separate out serum. Brains were collected following perfusion, halved along the sagittal midline, then frozen. All brains were visually inspected for signs of blood during collection. In the occasional cases where brain perfusion was incomplete, serum and brain tissue from those animals was excluded from all further analysis.

Levels of bumetanide in serum and brain were analyzed by LC-MS/MS, using a previously described method. [23] The lower limit of quantitation for the assay was 1 ng/ml with an accuracy of 95%. [23] Briefly, 15 µl of serum were mixed with 135 µl of internal standard (0.2 µM D_5_-bumetanide in acetonitrile), then vortexed for 1 min to precipitate protein, and centrifuged for 5 min at 16,200 relative centrifugal force. For brain samples, tissue from one hemisphere (∼0.5 g) was homogenized in 270 µl of internal standard and additional acetonitrile, then centrifuged for 10 min at 16,200 relative centrifugal force. Supernatant was analyzed using a Waters Quattro Premier mass spectrometer equipped with an electrospray ionization source, and a Waters Acquity UPLC system (Waters Corp., MA) with an Atlantis T3 column (Waters Corp., 100 × 2.1 mm, 3 µm). Selected reaction monitoring (SRM) transitions were monitored for both bumetanide and D_5_-bumetanide. Additionally, the ratio of bumetanide concentration in brain to that in serum was calculated for each animal as a relative measure of drug elimination rates from the brain and serum. Of the 51 rats tested, three (2 control, 1 hypoxic) had brain levels below the assay’s lower limit of quantitation (<0.01 ng/g) [23] and were therefore excluded from the analysis.

### Assessment of Constitutive Cell Death After Drug Administration

Many commonly used antiepileptic drugs, including high doses of phenobarbital, acutely increase constitutive apoptosis in the developing rodent brain following a single dose, [24] yet Bittigau et al previously demonstrated that phenobarbital doses below 40 mg/kg did not induce constitutive apoptosis. [26] We tested the effects of the aforementioned dosing of phenobarbital (15 mg/kg) and bumetanide (0.3 mg/kg), either alone or in combination, on constitutive cell death. A vehicle-treated rat was used as a negative control, and a rat treated with a single 1 mg/kg i.p. dose of the NMDA receptor antagonist, dizocilpine (MK-801), which has previously been shown to increase constitutive apoptosis, [27] was used as a positive control. At 48 hr after treatment, rats were anesthetized (100 mg/kg pentobarbital) and perfused transcardially with PBS, followed by 4% paraformaldehyde. Brains were removed and post-fixed overnight with 4% paraformaldehyde in PBS, and then cryoprotected in 30% sucrose in PBS. 16 µm coronal sections were cut on a cryostat (Leica Microsystems, Germany), collected onto slides, and stored at −20°C until needed. Constitutive cell death was determined by terminal deoxynucleotidyl transferase-mediated dUTP nick-end labeling (TUNEL) with an anti-fluorescein antibody conjugated with a peroxidase reporter enzyme (Roche, IN), and visualized with 3,3′-Diaminobenzidine (DAB, Vector Laboratories Inc., CA). Sections were lightly counterstained with Methyl Green to enhance tissue visualization, then rapidly dehydrated through to 100% ethanol prior to clearing in Histoclear, and coverslipping with DPX mounting medium (Fluka Biochemika, Switzerland).

Coronal sections in the region of the mid-dorsal hippocampus (2.8–3.1 mm from Bregma, 2.6–3.0 mm lateral to midline) [28] were examined, allowing assessment of multiple brain regions in one section. Transmitted light images were obtained on a Zeiss Axioscope microscope, using a Spot digital camera and Advanced 4.5 software (Spot Imaging Solutions, MI). Non-overlapping low power photomicrographs were taken of parietal cortex, hippocampus, thalamus, caudate-putamen, and amygdala. The total area examined for each animal varied by region from about 1.7 mm^2^ for caudate putamen to over 4 mm^2^ for thalamus. Brain regions were analyzed separately and as a combined area (15 mm^2^) for the number of TUNEL-positive cells. TUNEL positive cells were evident as darkly stained pyknotic nuclei within a background of healthy appearing cells counterstained with methyl green. Parietal cortex from a rat treated with MK-801 was used as a positive control.

### Western Blot Analysis of NKCC1 and KCC2 Expression Following Seizures

Cortex and hippocampal tissue from rats sacrificed at 1, 12, 24, 48, and 168 hr following HS was dissected over cold PBS and membrane protein fractions were obtained, as previously described. [29] Naïve littermate rats that had not been exposed to hypoxia were used as controls. Briefly, tissue samples were homogenized over ice in a sucrose buffer containing one Complete Mini, Ethylenediaminetetraacetic acid (EDTA)-free protease inhibitor cocktail tablet (Roche) per 10 ml of buffer. Membrane fractions were collected and resuspended in lysis buffer. Total protein amounts were measured using the Bradford protein assay (Bio-Rad, CA), and samples were diluted for equal amounts of protein in each sample. Samples were run on 4–20% gradient Tris-HCl gels (Bio-Rad) and transferred onto polyvinylidene difluoride (PVDF) membranes via a semi-wet system (Invitrogen iBlot, Life Technologies Corp, NY). Membranes were blocked for 1 hr in 5% non-fat dry milk in Tris-buffered saline with 0.1% Tween 20 (Boston Bioproducts) before overnight incubation in primary antibody (NKCC1 (T4) at 1∶1000, Hybridoma Bank; KCC2 at 1∶500, Upstate; actin at 1∶10000, Invitrogen). Blots were then incubated for 1 hr in horseradish-peroxidase conjugated secondary antibodies (0.1 g/ml anti-rabbit IgG, 0.5 g/ml anti-mouse IgG, Invitrogen). Protein bands were visualized by chemiluminescence using the Image Reader LAS 3000 digital system and Image Gauge 4.0 software (Fuji Film Life Sciences, Japan). The optical densities (OD) for NKCC1 and KCC2 were first normalized to actin, averaged for each time point, and then expressed as a percentage of the control mean values from samples run on the same blot. We also calculated the ratio of NKCC1 to KCC2 from the normalized individual values (% of control means), as a measure of hypoxia-induced changes from normal expression patterns.

### Perforated Patch Clamp Recordings from Hippocampal Slices

Hippocampal slices were prepared at 24 hr after HS as previously described. [30], [31] Briefly, rat pups were decapitated 24 hr after HS. Littermate rats that had not been exposed to hypoxia were used as normoxic controls. Brains were rapidly removed and dissected in oxygenated (95% O_2_–5% CO_2_), ice-cold (2–5°C) cutting solution containing 210 mM sucrose, 2.5 mM KCl, 1.02 mM NaH_2_PO_4_, 0.5 mM CaCl_2_, 10 mM MgSO_4_, 26.19 mM NaHCO_3_, and 10 mM D-glucose (pH 7.4). Coronal hippocampal slices were sectioned at 300 µm from the middle third of the hippocampus using a vibratome (Lecia VT1000S, Leica). For electrophysiological recording, slices were transferred to a chamber containing oxygenated artificial cerebral spinal fluid (ACSF) containing 124 mM NaCl, 5 mM KCl, 1.25 mM NaH_2_PO_4_, 2 mM CaCl_2_, 1.2 mM MgSO_4_, 26 mM NaHCO_3_, and 10 mM glucose (pH 7.4 ) at 35°C for 30 min and thereafter at room temperature for at least 1 hr before use.

Gramicidin perforated patch clamp recordings were used in order to maintain the endogenous intracellular Cl^−^ concentration, as per our previously published protocol. [32] Electrodes for perforated-patch clamp recordings were prepared from thin-walled glass pipettes using a model P-87 micropipette puller (Sutter Instrument Co., CA). The electrode was filled with a KCl-based internal solution containing 150 mM KCl, and 10 mM HEPES (pH 7.2, adjusted with potassium hydroxide, 280–290 mOsm/kg). Gramicidin was dissolved in DMSO at 40 mg/ml, and then added to the pipette solution to a final concentration of 80 mg/ml. Electrodes were back filled with gramicidin-containing solution, then tip filled with gramicidin-free solution. The resistance of the patch electrode ranged from 3 to 6 MΩ. Perforated-patch recordings were made using an Axopatch 200A amplifier (Axon Instruments, CA) with a sampling frequency of 10 kHz after low-pass filtering at 1 kHz, using pClamp9 software (Clampex, Molecular Devices, CA). After the cell-attached formation, series resistance was monitored with 5 mV hyperpolarizing pulses. It typically took about 30 min for the series resistance to drop to 30–70 MΩ and stabilize.

GABA (100 µM) was briefly (100–200 ms) puffed onto the dendrites of the recorded neurons via a patch-type electrode using a valve-controlled pressure application system (Picospritzer II; Parker Hannifin Corporation, OH). We allowed at least 25 s between GABA applications. GABA_A_ receptor–mediated currents were pharmacologically isolated by application of 10 µM NBQX, and 100 µM DL-AP5 to block AMPA and NMDA receptors. The reversal potentials for the GABA-induced currents (E_GABA_) were determined by varying the holding potential of the recorded neuron in 10 mV increments, and then measuring peak amplitude of the GABA-activated currents. To parallel the in vivo treatment, phenobarbital (100 µM) and bumetanide (10 µM) were applied alone and in combination to test their effects on E_GABA_. Data were collected 10 min after drugs were applied, and each drug was applied for a total of 15 to 20 min. Recordings for each condition were compared to control recordings obtained prior to drug application. Current-voltage curves and data analysis were performed using Clampfit 9 (Molecular Devices).

### Statistical Analysis

Group measures are expressed as mean ± standard error of the mean (SEM); error bars also indicate SEM. Statistical analysis for differences between group means was performed using *t*-test and one-way analysis of variance (ANOVA) for multiple group comparisons.

## Results

### Acute Hypoxia-induced Behavioral and EEG Seizures

**Figure 2:**
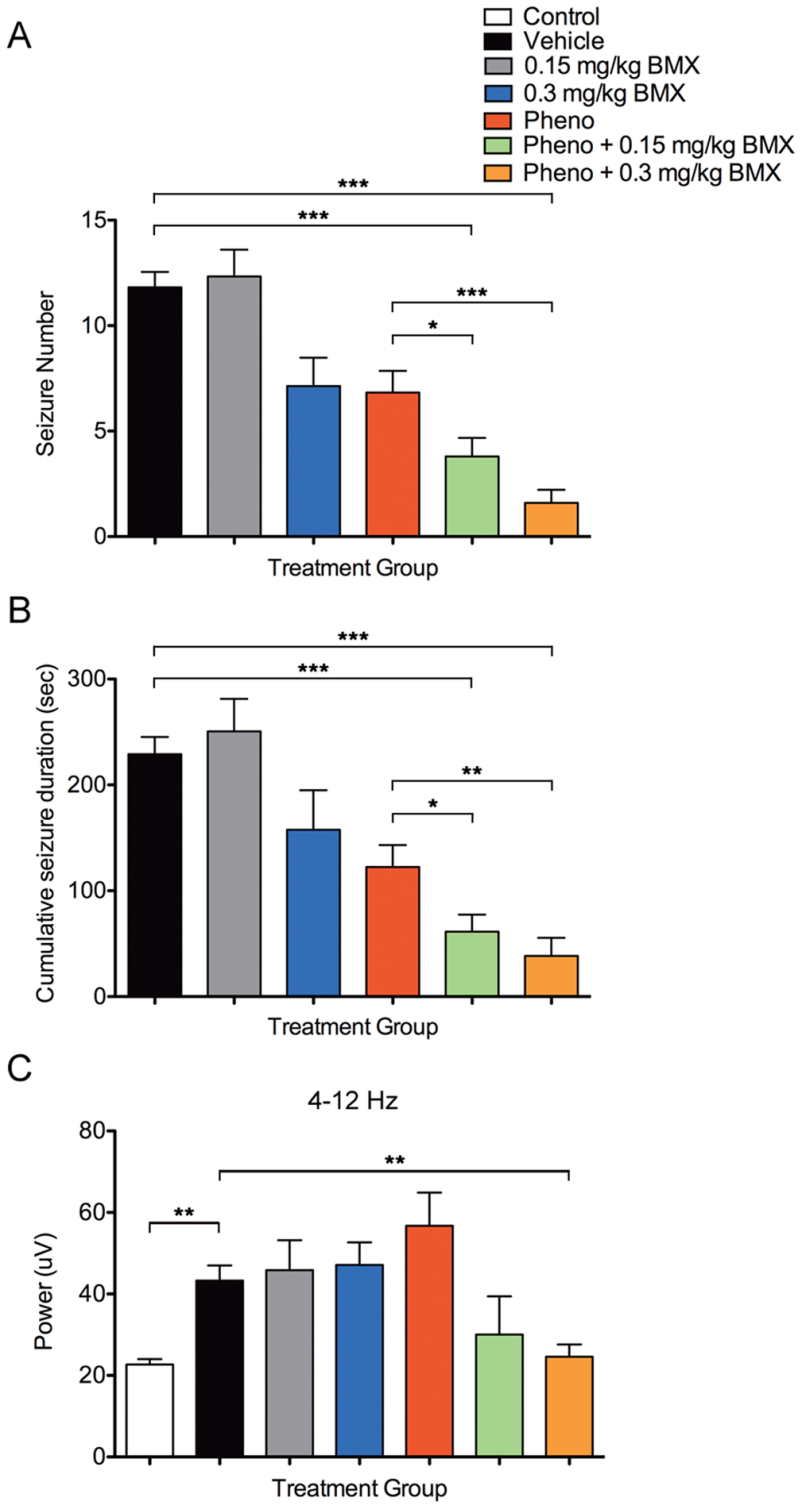
Seizure number, cumulative duration, and EEG power attenuation in rats treated with phenobarbital and bumetanide. The number of hypoxia-induced seizures (A), and cumulative seizure duration (B) were plotted for each treatment group. Vehicle-treated rats (n = 38) averaged 11.8±0.7 seizures, with an average cumulative seizure duration of 229±16.3 s. Treatment with phenobarbital (pheno) reduced seizure incidence by approximately 50% (n = 33, 6.8±1.0, 122±20.8 s), but did not completely attenuate activity. The combination of phenobarbital and 0.15 mg/kg bumetanide (pheno +0.15 mg/kg BMX) reduced seizure incidence by approximately 75% compared to vehicles (n = 35, 3.8±0.9, 61.3±16.1 s), and was more effective than phenobarbital alone. The higher dose (pheno +0.3 mg/kg BMX) was even more effective, decreasing seizure activity by ∼85% from vehicles (n = 25, 1.6±0.6, 38.5±17.0 s), and ∼75% from phenobarbital. (C) FFT analysis revealed increased spectral power between 4–12 Hz during seizures (vehicle). The average summed power between 4–12 Hz was plotted for each treatment group. Treatment with phenobarbital (n = 5, 56.8±8.1 µV), bumetanide (low dose: n = 3, 45.84±7.4 µV; high dose: n = 5, 47.1±5.5 µV), and the combination of phenobarbital and low dose bumetanide (n = 3, 30.0±9.4 µV) had little effect on summed power at these frequencies. However, in rats treated with the high dose combination (n = 4, 24.6±3.0 µV) summed power was reduced to levels similar to pre-hypoxia baseline EEG recordings (control). Mean ± SEM. Error bars indicate SEM. *p<0.05, **p<0.01, ***p<0.001.

Consistent with previous studies, [15], [22] exposure to graded global hypoxia induced acute seizures in 95% of the vehicle-treated rats (38 of 40 rats ≥ 5 seizures). Seizures were characterized by head bobbing, wet dog shakes, and tonic-clonic head and limb movements. During the 15 min hypoxic episode, vehicle-treated rats averaged 11.8±0.7 seizures, with an average cumulative seizure duration of 229±16.3 s (Fig. 2A, B).

To confirm that the observed behavioral events were in fact ictal EEG activity, video-EEG and FFT analysis was performed in a subgroup of rats during hypoxia. Of the 201 seizures recorded from all treatment groups, 52.7% were between 3 and 15 s in duration, and 24.9% were 15–30 s in duration. Seizure duration ranged from 3–113 s, with an average duration of 21.8±1.48 s, and all seizures were accompanied by the behavioral automatisms described earlier. FFT analysis was performed on frequency bands 4–12 Hz and 12–32 Hz, however, significant changes were only seen in the 4–12 Hz band (12–32 Hz data not shown).

### Effect of Combination Treatment on Hypoxia-induced Behavioral Seizures and EEG Ictal Activity

Treatment with phenobarbital alone (n = 33) prevented seizures in only 23% during hypoxia (Fig. 2A, B). Compared to the vehicle-treated HS group (n = 38), phenobarbital reduced average seizure number from 11.8±0.7 to 6.8±1.0 (*p*<0.001), and cumulative seizure duration was reduced from 229±16.3 s to 122±20.8 s (*p*<0.001). Low dose bumetanide (0.15 mg/kg) alone had very little effect on seizures in our model (n = 18, 12.3±1.3, 251±30.8 s), and monotherapy with the higher dose (0.3 mg/kg, n = 15) only moderately attenuated seizure activity (7.1±1.4, p<0.01; 158±37.3 s). In contrast, the combination of phenobarbital with low dose bumetanide (n = 35) reduced seizure incidence by approximately 75% compared to vehicles (3.8±0.9, 61.3±16.1 s, *p*<0.001), while combination with the high dose further decreased seizure activity by 85% from vehicle-treatment (n = 25, 1.6±0.6, 38.5±17.0 s, *p*<0.001), and ∼75% from phenobarbital alone (*p*<0.01). The low and high dose combined treatments completely suppressed seizures in 46% and 64% of the rats treated, respectively.

FFT analysis was used to quantitate differences in ictal activity between treatment groups. Vehicle-treated rats (n = 4) showed increased spectral power between 4–12 Hz (summed power 43.3±3.7 µV) during seizures (Fig. 2C) compared to the baseline EEG epochs (22.7±1.3 µV, *p*<0.01). When administered alone, no significant effect on EEG power was seen with phenobarbital (n = 5, 56.8±8.1 µV), low dose bumetanide (n = 3, 45.84±7.4 µV), or high dose bumetanide (n = 5, 47.1±5.5 µV), as compared to vehicle. While combination of phenobarbital with low dose bumetanide appeared to have only a modest effect (n = 3, 30.0±9.4 µV), its combination with high dose bumetanide significantly reduced summed power compared to vehicle (n = 4, 24.6±3.0 µV, *p*<0.01), approximating levels seen in baseline EEG recordings.

### Effects of Phenobarbital and Bumetanide on Constitutive Cell Death during Development

**Figure 3:**
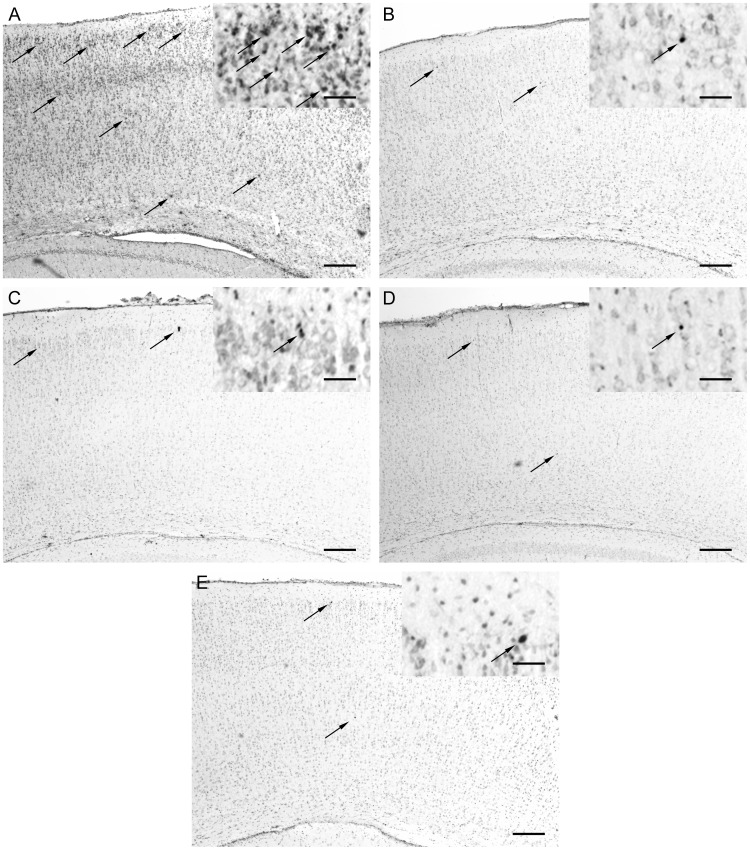
Phenobarbital and bumetanide, singly or in combination, did not increase constitutive cell death in P10 rat pups. Representative photomicrographs and magnified insets of TUNEL staining on 16 µm coronal sections of parietal cortex 48 h after treatment with (A) MK801 (positive control), (B) vehicle (DMSO in 0.9% saline) (n = 10), (C) phenobarbital (n = 11), (D) high dose bumetanide (n = 11), or (E) combined phenobarbital and high dose bumetanide (n = 8). Sections were lightly counterstained with methyl green. Pictures were taken at low power to maximize scoring, and magnified insets show detail. Other regions examined were amygdala, caudate-putamen, hippocampus, and thalamus. Apoptotic cells can be seen as dark pyknotic nuclei (arrows denote examples). No treatment significantly increased constitutive apoptosis compared to vehicle when analyzed within each region or in the total area (p = 0.129). A P7 rat pup treated with MK801 served as a positive control, and showed numerous apoptotic neurons clustered in layer II but also in other cortical layers. Scale bar = 200 µm (inset scale bar = 35 µm).

In vehicle-treated rats (n = 10) the distribution of TUNEL positive cells was sparse, consistent with prior reports, ranging between 1.4±0.14 to 1.9±0.21 cells/mm^2^ in 5 different anatomical regions: parietal cortex, amygdala, caudate putamen, hippocampus, and thalamus. [24], [27] Neither phenobarbital (n = 11) nor high dose bumetanide (n = 11), administered separately or in combination (n = 8), caused a significant increase in apoptosis above background levels seen in vehicle-treated parietal cortex (*p = *0.183), amygdala (*p = *0.727), caudate putamen (*p = *0.317), hippocampus (*p = *0.724), or thalamus (*p = *0.096) (Figure 3). Comparison of cumulative apoptosis within all 5 regions also revealed no alterations in apoptosis with the different drug treatments (*p* = 0.129).

### Temporal Profile of Serum and Brain Bumetanide Levels Following Treatment

**Figure 4:**
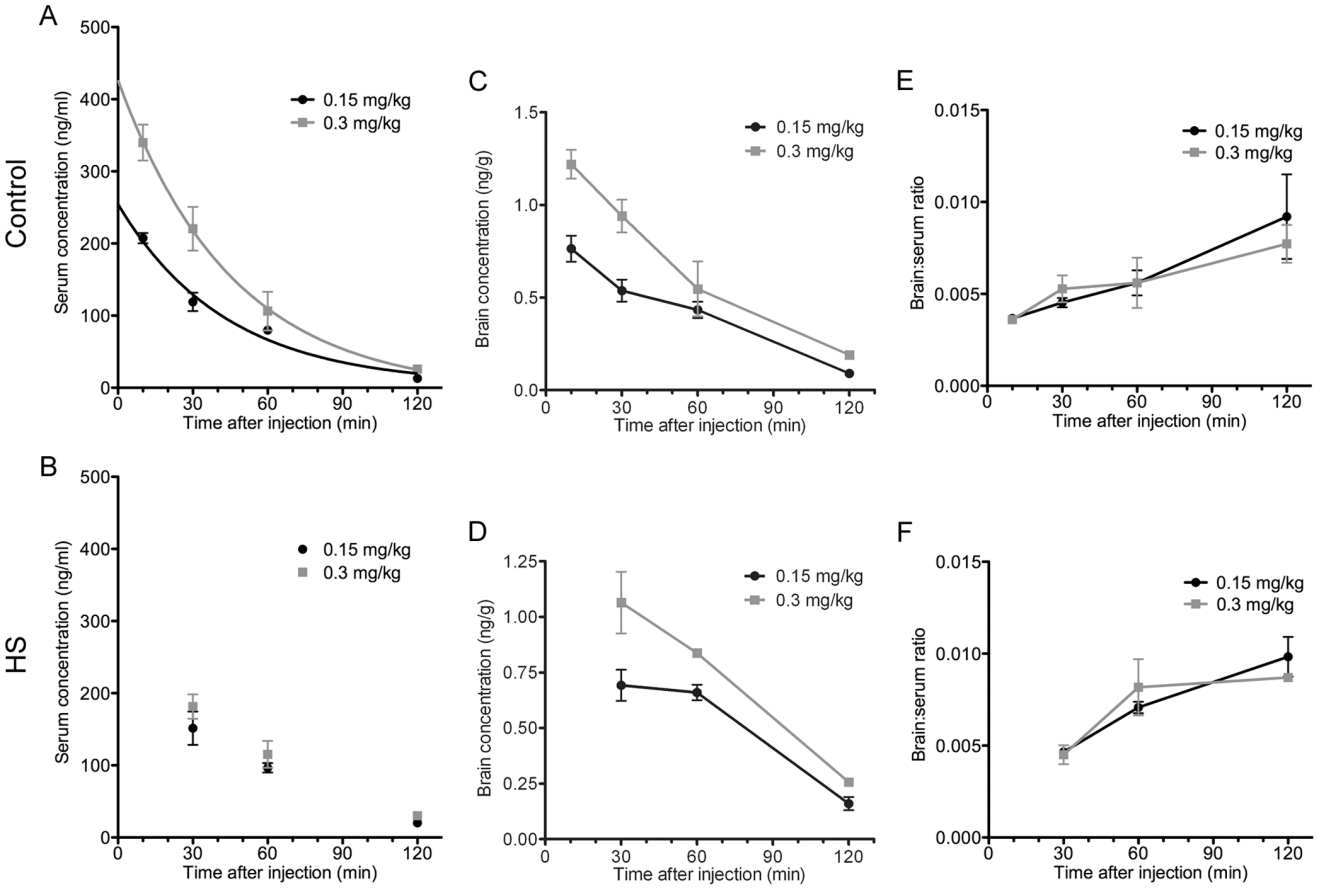
Bumetanide serum and brain levels, and brain:serum concentration ratios in control and hypoxic rats. Serum bumetanide levels in rats after hypoxia-induced seizures (HS, B) and in littermate controls (A) were plotted for the low (0.15 mg/kg) and high (0.3 mg/kg) bumetanide doses. In controls, average elimination half-lives of 29.9 min (low dose) and 31.6 min (high dose) were calculated using GraphPad Prism. Best-fit values were calculated assuming a one-phase exponential decay. Brain levels for control (C) and HS (D) rats were plotted for the low and high bumetanide doses. Plots of the brain:serum concentration ratios (E, F) show that bumetanide was eliminated more slowly from the brain than from serum. Mean ± SEM. Error bars indicate SEM.

Bumetanide serum and brain levels were determined in 51 rat pups (n = 28 controls, n = 23 HS, Tables S1, S2). Bumetanide was rapidly eliminated from serum (Fig. 4A, B), with an average half-life of 29.9 min at the low dose and 31.6 min at the high dose in controls, longer than the 10 min half-life reported in adult rats. [33] Average serum concentrations of 208±7.1 and 340±24.9 ng/ml were determined 10 min after injection (n = 3, 3) and fell to 13.1±2.7 and 25.8±4.0 ng/ml at 120 min (n = 2, 4) for the low and high dose, respectively. Similar levels were seen in treated HS rats, peaking at 152±23.1 and 182±16.9 ng/ml at 30 min (n = 4, 4) and falling to 20.1±5.4 and 30.2±1.5 ng/ml at 120 min (n = 3, 3) for the low and high dose, respectively. Due to the absence of the 10 min group in the hypoxic rats, however, half-lives could not be determined for this group as three data points was insufficient to obtain an accurate line fit.

While still above the lower limit of detection of the assay (0.1 ng/ml), [23] brain levels were very low, with peak levels in control pups at 0.76±0.07 and 1.22±0.08 ng/g 10 min after injection for the low and high dose, respectively (Fig. 4C, D). These brain levels were on average higher in rats exposed to hypoxia than in controls (data not shown), potentially indicating enhanced drug uptake due to an increase in blood brain barrier permeability or increased blood flow. Bumetanide appeared to be eliminated more slowly from the brain as evidenced by the brain:serum concentration ratio (Fig. 4E, F), which more than doubled in both control and hypoxic rats.

### Transient Changes in NKCC1 and KCC2 Expression Following Neonatal Seizures

**Figure 5:**
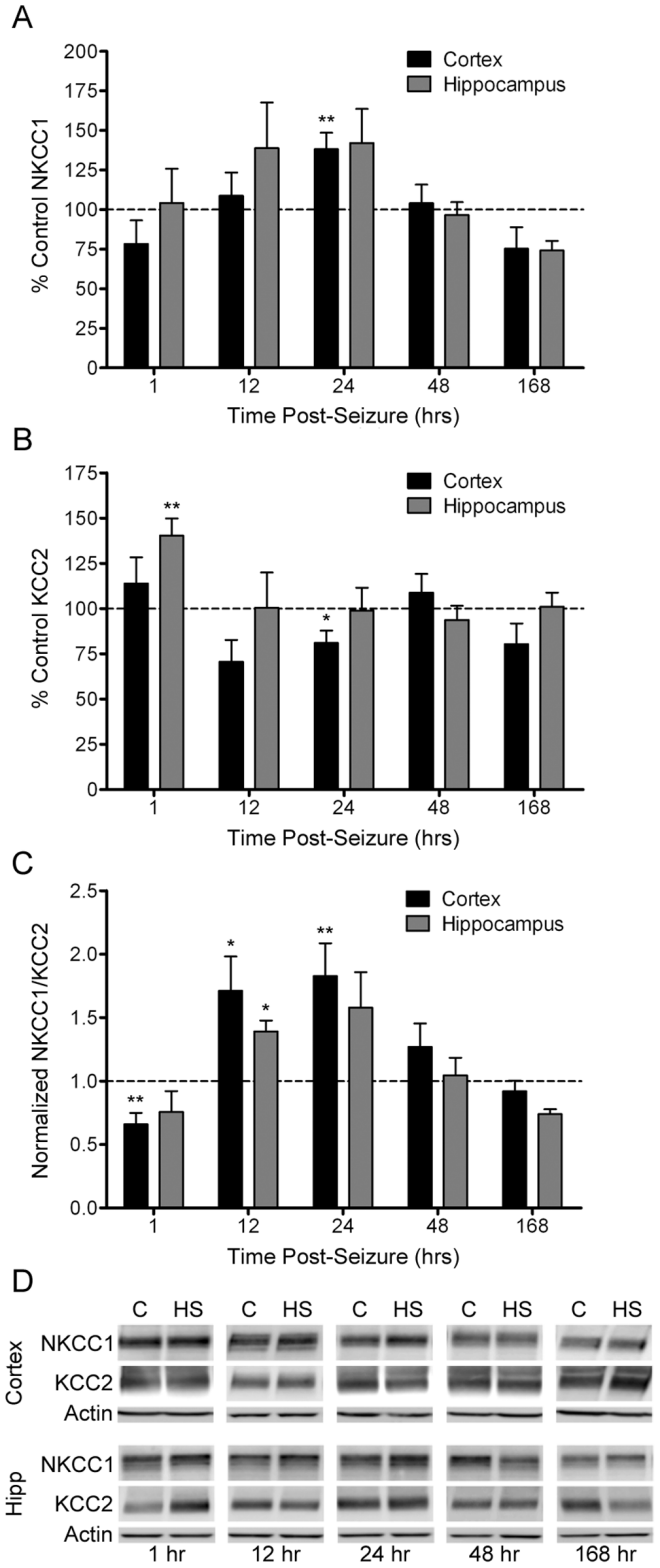
Seizure-induced alterations in NKCC1 and KCC2 protein levels. Using Western Blot, NKCC1 and KCC2 protein levels were assessed in cortical and hippocampal tissue obtained from P10 rats at 1, 12, 24, 48, and 168 hr after hypoxia-induced seizures (HS), and compared to age-matched littermate controls. Optical densities for NKCC1 and KCC2 were normalized to actin, averaged for each time point, and then the average expression in hypoxic animals was normalized to that in control animals. NKCC1 (A) and KCC2 (B) expression was plotted as a percent of control (dotted line). NKCC1 increased transiently at 24 hr post-HS (n = 8, 138±10.5% control, *p* = 0.005) in cortex, but showed no significant change in hippocampus. KCC2 expression increased early at 1 hr post-HS (n = 4, 140±9.6% control, *p* = 0.004) in hippocampus, with a later cortical increase 24 hr post-HS (n = 8, 81±6.7% control, *p* = 0.025). The normalized ratio of NKCC1 to KCC2 (C) was then calculated as a measure of changes in the relative expression of NKCC1 and KCC2 from normal expression patterns. In the cortex, the ratio of NKCC1 to KCC2 initially decreased 1 hr after seizures (n = 7, 0.66±0.09 fold, p = 0.003), and then increased at 12 and 24 hr (12 hr: n = 7, 1.71±0.27 fold, *p* = 0.046; 24 hr: n = 8, 1.83±0.26 fold, *p* = 0.008), as compared to controls. In the hippocampus, NKCC1/KCC2 was significantly higher at 12 hr (n = 8, 1.39±0.09 fold, p = 0.016), as compared to controls. (D) Representative western blot images for NKCC1, KCC2, and actin protein levels in cortex (top) and hippocampus (bottom). C = control; HS = hypoxic seizures. Mean ± SEM. Error bars indicate SEM.*p<0.05, **p<0.01.

The effects of hypoxic seizures on the relative expression of NKCC1 and KCC2 were assessed by western blotting in cortical and hippocampal tissue removed at 1, 12, 24, 48, and 168 hr post-HS. In the cortex, HS caused an initial decrease in the expression of NKCC1 relative to KCC2 (n = 7, 0.66±0.09 fold, *p* = 0.003), followed by a nearly two-fold increase between 12 and 24 hr post-hypoxia (12 hr: n = 7, 1.71±0.27 fold, *p* = 0.046; 24 hr: n = 8, 1.83±0.26 fold, *p* = 0.008) before stabilizing to levels similar to controls (Figure 5). In the hippocampus, the ratio of NKCC1 to KCC2 underwent a transient increase 12 hr after HS, rising 1.39±0.09 fold (n = 8, *p* = 0.016) before returning to normal levels.

### Phenobarbital and Bumetanide Reversed Depolarized E_GABA_ in CA1 Neurons from *ex vivo* Slices Removed at 24 hr post-HS

**Figure 6:**
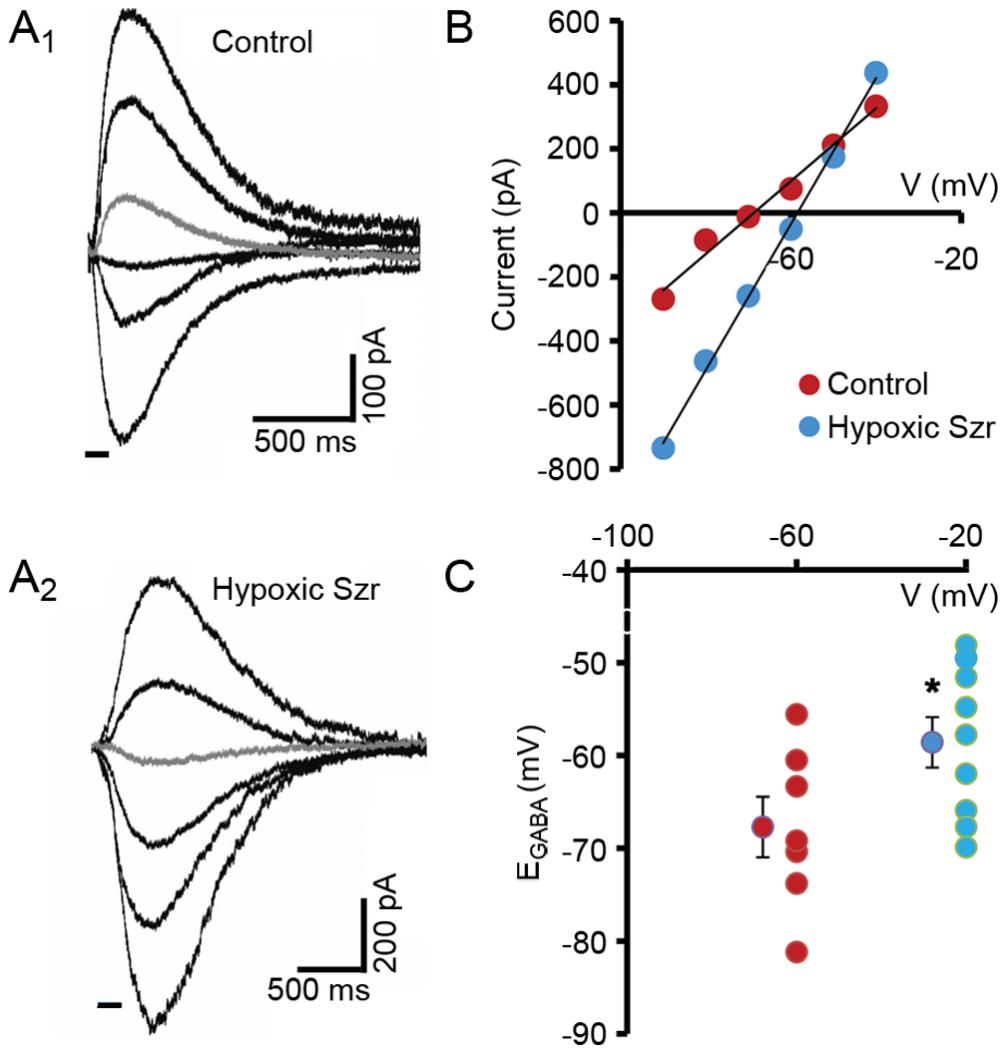
Hypoxic seizures induce a positive shift in E_GABA_ in hippocampal CA1 pyramidal neurons. Representative recordings of GABA-evoked currents in CA1 neurons from normoxic control hippocampal slices (A1) versus slices removed at 24 hr post-hypoxic seizures *in vivo* at P10 (A2). (B) Current–voltage curves for the recordings shown in A1 and 2. The data points were fitted with a straight line. (C) Summary of the reversal potential of the GABA-evoked currents in CA1 pyramidal neurons in slices removed after hypoxic seizures (n = 9, −58.58±2.72 mV) compared with control slices (n = 7, −67.70±3.26 mV). Error bars indicate SEM. *p<0.05.

**Figure 7:**
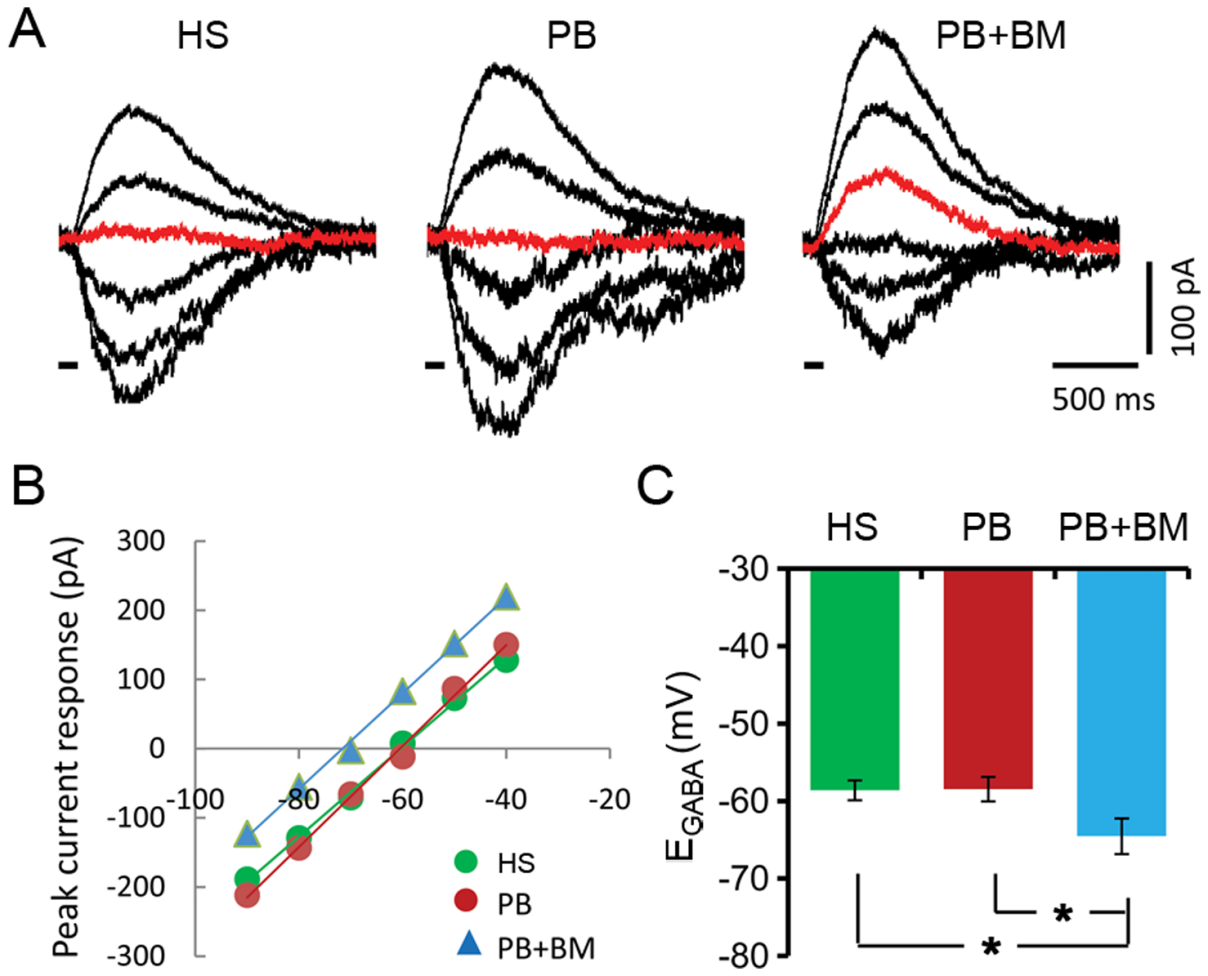
The combination of phenobarbital and bumetanide alter E_GABA_ in CA1 neurons from slices removed at 24 hr post-hypoxic seizures. GABA-evoked currents were measured from hippocampal slices taken from rats (n = 6) 24 hr after hypoxia-induced seizures. (A) Typical examples of GABA-evoked currents in CA1 neurons at various holding potentials after application of phenobarbital (PB, 100 mM), and phenobarbital in combination with bumetanide (PB+BM). Treatment effects were compared to normal conditions (HS) prior to drug administration. (B) Current–voltage curves for the recordings shown in A. The data points were fitted with a straight line. (C) Summary of the reversal potential of GABA-evoked currents under different recording conditions: control (HS, −58.61±1.28 mV), phenobarbital only (PB, −58.46±1.58 mV), and phenobarbital in combination with bumetanide (PB+BM, −64.53±2.30 mV). Error bars indicate SEM. *p<0.05.

CA1 hippocampal neurons in slices removed from rat pups 24 hr post-HS showed a significant positive shift in E_GABA_ (n = 9, −58.58±2.72 mV, Figure 6) as compared to control slices (n = 7, −67.70±3.26 mV, *p*<0.05), with no changes in resting membrane potential between the two groups (*p*>0.05). We next compared the effects of phenobarbital (100 µM) alone or in combination with bumetanide (10 µM) to control conditions (n = 6, Figure 7). Phenobarbital alone increased GABA-evoked current amplitude, but had no effect on GABA reversal potential (−58.46±1.58 mV) compared to control conditions (−58.61±1.28 mV, *p*>0.05). In contrast, the combination of phenobarbital with bumetanide significantly decreased reversal potential (−64.53±2.30 mV, *p*<0.05) to baseline values seen in naïve age-matched littermates (*p*>0.05, Fig. 6A1). These data suggest GABA-mediated bumetanide-sensitive excitation in neurons post-seizure.

## Discussion

The present study provides preclinical support for the use of bumetanide as an add-on therapy in the treatment of neonatal seizures. Similar to human neonates, the second postnatal week of life in the rat is characterized by high expression of NKCC1 in cortical and hippocampal neurons, which could contribute to resistance to GABA agonist anticonvulsants. [17] In a clinically relevant model of hypoxia-induced seizures, the combination of the NKCC1 inhibitor, bumetanide, and phenobarbital showed superior efficacy to phenobarbital alone. Systemic administration of bumetanide resulted in a dose-dependent increase in both serum and brain concentrations. Seizures in the immature rat transiently increased the NKCC1:KCC2 ratio, and hippocampal slices prepared from rats following seizures exhibited a shift in GABA reversal potential consistent with NKCC1-mediated Cl^−^ intracellular flux, which was reversed by combined application of phenobarbital and bumetanide. Unlike other AEDs, the combination of bumetanide and low dose phenobarbital used here does not appear to increase constitutive apoptosis during the second postnatal week, [24], [26], [27], [34] suggesting that combination therapy may provide more efficacy and safety than high dose phenobarbital alone.

### Neonatal Seizure Therapies

The standard of care for neonatal seizures is treatment with high doses of phenobarbital and/or phenytoin, yet studies have failed to show significant efficacy. [2], [6], [35] Although seizures are often only present for a few days postnatally in the case of birth asphyxia and HIE, the consequences of neonatal seizures can be severe. [6], [36] Unique age-specific mechanisms of seizure generation in the newborn brain are likely to underlie the refractory nature of neonatal seizures, [37] and include the heightened expression of excitatory glutamate neurotransmitter receptors, and a relative under-expression of inhibitory GABA receptors combined with low GABA synthesis. [6] While agents that inhibit the AMPA subtype of the glutamate receptor, such as topiramate or talampanel, are protective in neonatal seizure and hypoxia/ischemia models, [5], [38]–[40] the use of these agents has yet to be effectively translated to clinical trials due to their lack of parenteral formulations that are necessary in these critically ill neonates.

The NKCC1 inhibitor, bumetanide, is FDA approved for use as a diuretic and has been used in neonates safely for decades for this indication. [20], [21] Most recently, a Phase I/II dose escalation study of bumetanide (0.15, 0.25 or 0.3 mg/kg) as an add-on to phenobarbital in infants with neonatal seizures (clinicaltrials.gov, NCT00830531) is being conducted to more specifically address bumetanide safety in its use as an AED. The population of this safety study are infants with HIE or stroke, and hence the present preclinical study was performed to examine efficacy of bumetanide in HS in rodents to more closely resemble the human disease state. While several studies have demonstrated efficacy of bumetanide in chemoconvulsant models of seizures in immature rats as well as in *in vitro* hippocampal preparations, [16], [17] its efficacy and safety alone or in combination with phenobarbital have never been tested in a clinically relevant model of HIE-induced seizures, nor has there been any direct assessment of bumetanide levels in serum and brain.

### Efficacy of Combination Therapy with Phenobarbital and Bumetanide

The present study shows that phenobarbital alone, at a dose effective in adult rodents, [41] has only modest anticonvulsant activity in our neonatal rat model of HS. This is consistent with the limited efficacy seen in human studies. [35] Addition of the 0.15 mg/kg low bumetanide dose used in prior studies [17], however, resulted in a significant improvement, while addition of higher doses of 0.3 mg/kg resulted in almost complete attenuation of seizure activity. In contrast to prior reports, [17] [42] bumetanide alone had little to no efficacy in this model, suggesting that the relative role of basal NKCC1 expression may differentially regulate the model-dependent seizure susceptibility. Indeed the current Phase 1 trial is that of a combination of bumetanide with phenobarbital (clinicaltrials.gov, NCT00830531). Previous studies have demonstrated that behavioral seizures and EEG changes are correlated with an increase in EEG power, [15], [43], [44] which can be reduced with certain anticonvulsants. [15], [44] In our study, FFT analysis revealed a similar correlation between EEG power and seizure frequency. Neither phenobarbital alone nor bumetanide had any effect on EEG power, however, the combination of phenobarbital and 0.3 mg/kg bumetanide was able to reduce power to levels resembling baseline recordings. Taken together, these results support our prior reports on kainate seizures in neonatal rats, [17] and suggest that phenobarbital in combination with bumetanide, especially at the higher dose, exhibits an acute synergistic effect on seizures.

While the present study demonstrates an acute effect on symptomatic hypoxic seizures, future studies will need to address whether these doses alter long-term cognitive and epilepsy outcomes. We have recently shown that other agents that suppress activity-dependent signaling administered around the seizure can prevent long-term epilepsy and also autistic-like behavior seen in this model. [45] It is possible that acute seizure suppression with bumetanide and phenobarbital may have comparable effects either due to a direct role of NKCC1 in brain maturation, or secondary to more non-specific seizure suppressing effects. Indeed, a recent report showed that bumetanide in combination with phenobarbital, when used in addition to hypothermia, shows superior neuroprotective efficacy for acute and long term outcomes in a rodent neonatal stroke model. [46] Additionally, recent reports indicate that long-term treatment may decrease autistic behavior in human infants without side effects. [47] These reports suggest a more expansive role for bumetanide in treating the long-term consequences of neonatal seizures, which we hope to investigate further in future studies.

### Evidence for Enhanced NKCC1 Expression and Functional Depolarizing GABA Currents in Hypoxia-induced Seizures

The relative enhanced expression of NKCC1 in the immature rodent and human brain as compared to adult [10], [17], [48] has been thought to contribute to enhanced seizure susceptibility and the refractoriness of neonatal seizures to GABA agonists, such as phenobarbital. [49] Neuronal activity can modulate NKCC1 expression and consequent E_GABA_, [50] and seizure-induced NKCC1 expression following status epilepticus [51] has been postulated to contribute to epileptogenesis in adult rats. [33] Here we show that NKCC1 is transiently increased in hippocampus and cortex within the first 24 hr post-seizure, suggesting that seizures may further increase the level of GABA_A_ receptor mediated depolarization. NKCC1 can be upregulated both transcriptionally following excitotoxic insults and post-translationally through phosphorylation at its Thr-212 and Thr-217 sites on the cytoplasmic N terminus. [52].

In intact hippocampi prepared from immature rats or mice following chemoconvulsant-induced seizures, intracellular Cl^−^ progressively increases over a matter of hours, with a reversal of GABA_A_ receptor-mediated currents, a simultaneous increase in NKCC1 neuronal membrane expression, and a progressive decrease in the efficacy of phenobarbital. [50] Indeed, here we show that CA1 neurons in hippocampal slices removed from rats at 24 hr post-HS exhibit a shift in GABA reversal potential that can be reversed with application of phenobarbital and bumetanide. Likewise, recurrent kainate-induced seizures in immature rat brain have been shown to be associated with progressive increases in NKCC1 activity, [50] suggesting that this shift may be a generalized response in immature neurons.

### Dose-dependent Increases in Brain and Serum Bumetanide Levels in Control and Hypoxic Neonatal Mice

As the bumetanide doses in this rat model are similar to those being used in humans in the Phase I/II pharmacokinetic and safety study, an evaluation of pharmacokinetics was justified in the rat model using the same assay as in the human trial. [23] We show here that bumetanide levels are indeed detectable in both brain and blood following i.p. administration, and that the half-life in serum is approximately 20 min longer than the average half-life of the drug in adult rats for both doses. [33] Serum levels obtained here in the P10 rat with 0.15 and 0.3 mg/kg were in the 200–350 ng/ml range (within the diuretic range in human infants) [53], while plasma levels reported in the adult following much higher 15 mg/kg doses were only 27 µg/ml. [33] In adult animals, peak brain levels with this higher dose of bumetanide (15 mg/kg) were in the 0.1–0.3 µg/g range, and when lower doses were used the brain levels were below the detection threshold (0.1 µg/g) for the assay used. [33] Here, we employed a more sensitive LC-MS/MS assay with a lower detection range, [23] which was required since in the present study the administered doses were over ten-fold lower than those used by Brandt, et al. [33] We showed brain levels following the 0.3 mg/kg dose peaked at 1.2 ng/ml in P10 control rats, with a trend towards slightly higher brain concentrations in the HS rats, suggesting a seizure-associated increase in blood brain barrier permeability or increased blood flow. These results suggest only a modest age-dependent difference in bumetanide brain penetration, [54] as adult rats dosed with 0.3 mg/kg result in brain:serum ratios of approximately 0.007, [23] comparable to the 0.007–0.009 ratio at 0.3 mg/kg dose we report in P10 rats.

### Lack of Adverse Effects on Constitutive Apoptosis with Bumetanide Add-on Therapy

GABA agonists such as phenobarbital (at doses higher than 40 mg/kg [26]) and benzodiazepines, NMDA receptor antagonists, and the anticonvulsants including phenytoin, valproate, and vigabatrin, can increase constitutive neuronal apoptosis when the agents are delivered to immature rodents within the first two weeks of life. [24], [27], [34] However, administration of other anticonvulsants such as topiramate and levetiracetam do not appear to elicit these same side effects. [55], [56] Similarly, we found no increase in apoptotic neurons following administration of 15 mg/kg phenobarbital and 0.3 mg/kg bumetanide, either alone or in combination, to P10 rats. Interestingly, daily bumetanide treatment from P7–P14 (the age range in the present study and analogous to term human) [7] did not cause developmental delays in neurotransmission or alterations in anxiety or startle behavior, although treatment prior to P7 did cause adverse effects. [37].

### Conclusion

In light of the fact that bumetanide is currently under investigation as an add-on therapy to phenobarbital in a neonatal seizure Phase I/II trial, we show that doses of bumetanide similar to those currently being tested in our human trial were indeed effective in combination with phenobarbital in a rat model of neonatal seizures, without any increases in neuronal apoptosis. Effective doses were associated with a serum concentration of 100–300 ng/ml, within the range previously shown to be tolerated by human infants when administered for diuretic purposes. [20] These findings provide preclinical translational data to support ongoing clinical trials with bumetanide in neonatal seizures.

## Supporting Information

Table S1 **Serum and brain bumetanide levels, and brain:serum ratio in littermate controls.** Dose = bumetanide dosage. Time = min after bumetanide injection. n = number of rats. Concentrations (Conc.) are expressed as mean ± SEM.
(DOCX)

Table S2 **Serum and brain bumetanide levels, and brain:serum ratio after hypoxic seizures.** Dose = bumetanide dosage. Time = min after bumetanide injection. n = number of rats. Concentrations (Conc.) are expressed as mean ± SEM.
(DOCX)

Methods S1. (DOCX)
