# Correction: Bumetanide Enhances Phenobarbital Efficacy in a Rat Model of Hypoxic Neonatal Seizures

**DOI:** 10.1371/annotation/48a011e6-e4d0-4706-9a28-857eba8cfb31

**Published:** 2013-08-13

**Authors:** Ryan T. Cleary, Hongyu Sun, Thanhthao Huynh, Simon M. Manning, Yijun Li, Alexander Rotenberg, Delia M. Talos, Kristopher T. Kahle, Michele Jackson, Sanjay N. Rakhade, Gerard T. Berry, Frances E. Jensen

The name of the 11th author was incorrectly given. The correct name is: Gerard T. Berry. 

The correct abbreviation in Contributions is: GTB. 

In addition, an affiliation is missing for the 11th author. In addition to that affiliation already indicated for Gerard T. Berry, this author is affiliated with the following institution: Harvard Medical School, Boston, Massachusetts, United States of America.

