# Supplementary material for: Bumetanide Enhances Phenobarbital Efficacy in a Rat Model of Hypoxic Neonatal Seizures
Source: PLoS One. 2013 Mar 11;8(3):e57148. doi: 10.1371/journal.pone.0057148 (PMC3594228; doi:10.1371/journal.pone.0057148)
Supplement: Table S1 — Serum and brain bumetanide levels, and brain:serum ratio in littermate controls. Dose = bumetanide dosage. Time = min after bumetanide injection. n = number of rats. Concentrations (Conc.) are expressed as mean ± SEM. (DOCX) [file pone.0057148.s001.docx]

**Table S1: Serum and brain bumetanide levels, and brain:serum ratio in littermate controls**

| Dose (mg/kg) | Time (min) | n | Serum Conc. (ng/g) | Brain Conc. (ng/g) | Brain:serum ratio |
| --- | --- | --- | --- | --- | --- |
| 0.15 | 10 | 3 | 208 ± 7.1 | 0.76 ± 0.07 | 0.0037 ± 0.0002 |
| 0.3 | 10 | 3 | 340 ± 24.9 | 1.22 ± 0.08 | 0.0036 ± 0.0001 |
| 0.15 | 30 | 4 | 119 ± 12.8 | 0.54 ± 0.06 | 0.0045 ± 0.0003 |
| 0.3 | 30 | 4 | 220 ± 30.2 | 0.94 ± 0.09 | 0.0053 ± 0.0007 |
| 0.15 | 60 | 3 | 79.9 ± 4.2 | 0.43 ± 0.04 | 0.0056 ± 0.0007 |
| 0.3 | 60 | 3 | 106 ± 26.8 | 0.55 ± 0.15 | 0.0056 ± 0.0014 |
| 0.15 | 120 | 2 | 13.1 ± 2.7 | 0.12 ± 0.01 | 0.0092 ± 0.0023 |
| 0.3 | 120 | 4 | 25.8 ± 4.0 | 0.20 ± 0.01 | 0.0077 ± 0.0010 |
