# Supplementary material for: Bumetanide Enhances Phenobarbital Efficacy in a Rat Model of Hypoxic Neonatal Seizures
Source: PLoS One. 2013 Mar 11;8(3):e57148. doi: 10.1371/journal.pone.0057148 (PMC3594228; doi:10.1371/journal.pone.0057148)
Supplement: Table S2 — Serum and brain bumetanide levels, and brain:serum ratio after hypoxic seizures. Dose = bumetanide dosage. Time = min after bumetanide injection. n = number of rats. Concentrations (Conc.) are expressed as mean ± SEM. (DOCX) [file pone.0057148.s002.docx]

**Table S2: Serum and brain bumetanide levels, and brain:serum ratio after hypoxic seizures**

| Dose (mg/kg) | Time (min) | n | Serum Conc. (ng/g) | Brain Conc. (ng/g) | Brain:serum ratio |
| --- | --- | --- | --- | --- | --- |
| 0.15 | 10 |  | *N/A* | *N/A* | *N/A* |
| 0.3 | 10 |  | *N/A* | *N/A* | *N/A* |
| 0.15 | 30 | 4 | 152 ± 23.1 | 0.69 ± 0.07 | 0.0047 ± 0.0002 |
| 0.3 | 30 | 4 | 182 ± 16.9 | 1.07 ± 0.14 | 0.0045 ± 0.0005 |
| 0.15 | 60 | 4 | 96.6 ± 6.6 | 0.66 ± 0.04 | 0.007 ± 0.0003 |
| 0.3 | 60 | 4 | 115 ± 18.6 | 0.84 ± 0.02 | 0.008 ± 0.0015 |
| 0.15 | 120 | 3 | 20.1 ± 5.4 | 0.19 ± 0.04 | 0.010 ± 0.0011 |
| 0.3 | 120 | 3 | 30.2 ± 1.5 | 0.24 ± 0.01 | 0.009 ± 0.0002 |
