# Supplementary material for: Bumetanide Enhances Phenobarbital Efficacy in a Rat Model of Hypoxic Neonatal Seizures
Source: PLoS One. 2013 Mar 11;8(3):e57148. doi: 10.1371/journal.pone.0057148 (PMC3594228; doi:10.1371/journal.pone.0057148)
Supplement: Methods S1. — (DOCX) [file pone.0057148.s003.docx]

**Supplementary Methods**

Hypoxia induction: Briefly, oxygen concentration was maintained at 7% for 8 minutes, 5% for 6 minutes, and 4% for 1 minute before termination of hypoxia. Each animal was scored for the number of tonic-clonic seizures during the 15-minute hypoxic episode. Littermate controls were separated from their dams and kept at room air for the duration of the experiment. For all groups, normothermic body temperature was maintained at 32-34º C with a circulating water heater. All rats were returned to their dams within two hours.

Drug administration: Phenobarbital was diluted in 0.9% saline, and bumetanide (MP Biomedical) was dissolved in DMSO, then diluted in 0.9% saline. Vehicle solutions were 0.9% saline for phenobarbital, and DMSO diluted in 0.9% saline for bumetanide. Five treatment groups and one vehicle group were tested: phenobarbital alone, bumetanide alone (low or high dose), and phenobarbital in combination with either of the two bumetanide doses. Rats in the phenobarbital only and bumetanide only groups received an additional vehicle i.p. injection (DMSO in 0.9% NaCl or 0.9% NaCl) of the same volume they would have received if they were in a combined treatment group. Vehicle-treated rats received two vehicle i.p. injections (DMSO in 9% NaCl and 0.9% NaCl) of the same volume as those in the combined treatment group.

Video EEG recordings and analysis: A reference contact was positioned over the dorsal snout at midline, with two active contacts in the scalp over the parietal regions bilaterally. A fourth electrode was placed in the skin of the torso to record electrocardiogram (EKG), and a ground electrode was placed in the lower back. SWE implantation causes only minimal, momentary pain, and is well tolerated by the rat pups.^1^ Video-EEG monitoring was performed in a Plexiglas recording chamber, and animals were allowed free movement around the recording chamber. The electrodes were connected via flexible cables to a commutator in the ceiling of the recording chamber. Signal from the electrode assembly was amplified (x10,000), digitized, and filtered using a digital 32-channel EEG-recording apparatus from Stellate Systems Inc. (Natus Medical Systems, CA).

For spectral analysis, time frequency analysis of EEG power was carried out by consecutive 10-second Fast-Fourier transform (FFT) analysis. EEG was filtered using 1 Hz high-pass and 60 Hz low-pass filtering to remove ambient noise, binned in 20 s intervals, and power within the epoch was determined. The summed power was then calculated for frequency bands 4-12 Hz, and 12-32 Hz.

Assay for bumetanide levels in serum and brain: An additional group of rats was also exposed to global hypoxia prior to sacrifice, using the treatment paradigm described earlier. The 10 min group could only be collected for control rats due to the treatment paradigm. Blood was collected by cardiac puncture before perfusion, then clotted to separate out serum. Brains were collected following perfusion, halved along the sagittal midline, then frozen. All brains were visually inspected for signs of blood during collection. In the occasional cases where brain perfusion was incomplete, serum and brain tissue from those animals was excluded from all further analysis.

Assessment of apoptosis: At 48 hours after treatment, rats were anesthetized (100 mg/kg pentobarbital) and perfused transcardially with PBS, followed by 4% paraformaldehyde. Brains were removed and post-fixed overnight with 4% paraformaldehyde in PBS (Boston Bioproducts, MA), and then cryoprotected in 30% sucrose in PBS. 16 μm coronal sections were cut on a cryostat (Leica Microsystems, Germany), collected onto slides (Fisherbrand Superfrost Plus, Thermo Fisher Scientific, MA), and stored at -20° C until needed. Constitutive cell death was determined by terminal deoxynucleotidyl transferase-mediated dUTP nick-end labeling (TUNEL) with an anti-fluorescein antibody conjugated with a peroxidase reporter enzyme (Roche, IN), and visualized with 3,3′-Diaminobenzidine (DAB, Vector Laboratories Inc., CA). Sections were lightly counterstained with Methyl Green to enhance tissue visualization, then rapidly dehydrated through to 100% ethanol prior to clearing in Histoclear, and coverslipping with DPX mounting medium (Fluka Biochemika, Switzerland). Coronal sections in the region of the mid-dorsal hippocampus (2.8-3.1 mm from Bregma, 2.6–3.0 mm lateral to midline )^2^ were examined, allowing assessment of multiple brain regions in one section. Transmitted light images were obtained on a Zeiss Axioscope microscope, using a Spot digital camera and Advanced 4.5 software (Spot Imaging Solutions, MI). Non-overlapping low power photomicrographs were taken of parietal cortex, hippocampus, thalamus, caudate-putamen, and amygdala. The total area examined for each animal varied by region from about 1.7 mm^2^ for caudate putamen to over 4 mm^2^ for thalamus. Brain regions were analyzed separately and as a combined area (15 mm^2^) for the number of TUNEL-positive cells. TUNEL positive cells were assessed 48 hr after drug administration and were evident as darkly stained pyknotic nuclei within a background of healthy appearing cells counterstained with methyl green. Parietal cortex from a rat treated with MK-801 was used as a positive control.

Measurement of brain and serum bumetanide levels. Levels of bumetanide in serum and brain were analyzed using LC-MS/MS, using a previously described method.^3^ The lower limit of quantitation for the assay was 1 ng/ml with an accuracy of 95%.^3^ Briefly, 15 μl of serum were mixed with 135 μl of internal standard (0.2 μM D_5_-bumetanide in acetonitrile), then vortexed for 1 min to precipitate protein, and centrifuged for 5 min at 16,200 relative centrifugal force (rcf). For brain samples, tissue from one hemisphere (~0.5 g) was homogenized in 270 μl of internal standard and additional acetonitrile, then centrifuged for 10 min at 16,200 rcf. Supernatant was analyzed using a Waters Quattro Premier mass spectrometer equipped with an electrospray ionization source, and a Waters Acquity UPLC system (Waters Corp., MA) with an Atlantis T3 column (Waters Corp., 100 x 2.1 mm, 3 μm). Selected reaction monitoring (SRM) transitions were monitored for both bumetanide and D_5_-bumetanide. Additionally, the ratio of bumetanide concentration in brain to that in serum was calculated for each animal as a relative measure of drug elimination rates from the brain and serum. Of the 51 rats tested, three (2 control, 1 hypoxic) had brain levels below the assay’s lower limit of quantitation (<0.01 ng/g)^3^ and were therefore excluded from the analysis.

Western blot analysis of NKCC1 and KCC2. Cortex and hippocampal tissue was dissected over cold PBS from rats sacrificed at 1, 12, 24, 48, and 168 hr following HS, and immediately flash frozen in cold absolute ethanol. Brain tissue dissected from aged-matched littermates that had not been exposed to hypoxia served as controls. Membrane protein preparations were obtained, as previously described.^4^ Briefly, tissue samples were homogenized over ice in a sucrose buffer containing one Complete Mini, Ethylenediaminetetraacetic acid (EDTA)-free protease inhibitor cocktail tablet (Roche) per 10 ml of buffer. Membrane fractions were collected and resuspended in lysis buffer. Total protein amounts were measured using the Bradford protein assay (Bio-Rad, CA), and samples were diluted for equal amounts of protein in each sample. Samples were run on 4-20% gradient Tris-HCl gels (Bio-Rad) and transferred onto polyvinylidene difluoride (PVDF) membranes via a semi-wet system (Invitrogen iBlot, Life Technologies Corp, NY). Membranes were blocked for 1 hr in 5% non-fat dry milk in Tris-buffered saline with 0.1% Tween 20 (Boston Bioproducts) before overnight incubation in primary antibody (NKCC1 (T4) 1:1000, Hybridoma Bank; KCC2 1:500, Upstate; actin 1:10000, Invitrogen). Blots were then incubated for 1 hr in horseradish-peroxidase conjugated secondary antibodies (0.1 g/ml anti-rabbit IgG, 0.5 g/ml anti-mouse IgG, Invitrogen). Protein bands were visualized by chemiluminescence using the Image Reader LAS 3000 digital system and Image Gauge 4.0 software (Fuji Film Life Sciences, Japan). The optical densities (OD) for NKCC1 and KCC2 were normalized to actin, averaged for each time point, and the group means for hypoxic rats were normalized to those in control rats for each antibody. We also calculated the ratio of NKCC1 to KCC2 from the normalized individual protein values as a measure of hypoxia-induced changes from normal expression patterns.

Hippocampal slice preparation: Hippocampal slices were prepared as previously described.^5, 6^ Briefly, rat pups were decapitated 24 hr after HS. Littermate rats that had not been exposed to hypoxia were used as normoxic controls. Brains were rapidly removed and dissected in oxygenated (95% O_2_–5% CO_2_), ice-cold (2-5° C) cutting solution containing 210 mM sucrose, 2.5 mM KCl, 1.02 mM NaH_2_PO_4_, 0.5 mM CaCl_2_, 10 mM MgSO_4_, 26.19 mM NaHCO_3_, and 10 mM D-glucose (pH 7.4). Coronal hippocampal slices were sectioned at 300 μm from the middle third of the hippocampus using a vibratome (Lecia VT1000S, Leica). For electrophysiological recording, slices were transferred to a chamber containing oxygenated “recording” artificial cerebral spinal fluid (ACSF) containing 124 mM NaCl, 5 mM KCl, 1.25 mM NaH_2_PO_4_, 2 mM CaCl_2_, 1.2 mM MgSO_4_, 26 mM NaHCO_3_, and 10 mM glucose (pH 7.4). Slices were incubated at 35°C for 30 min and thereafter at room temperature for at least 1 hr before use. In addition to Cl^­-^, bicarbonate (HCO_3_^-^) is also known to flux through the GABA_A_ channel and contribute to the GABA equilibrium potential (E_GABA_).^7, 8^ To avoid this problem, 4-(2-hydroxyethyl)-1-piperazineethanesulfonic acid (HEPES)-buffered ACSF is sometimes used to prevent HCO_3_^-^ flux through the GABA_A_ channel, thus bringing E_GABA_ very close to the Cl^-^ equilibrium potential (E_Cl_). For our experiments, however, we chose to use standard ACSF while measuring the E_GABA_, because at the relatively high levels of intracellular Cl- in this study HCO_3_^-^ has only a minor effect on E_GABA_.^9^

Perforated-patch clamp recordings: We chose to use perforated patch clamp recording in order to maintain the endogenous intracellular Cl^-^ concentration and avoid the time resolution problem associated with cell-attached recordings, using our previously published protocols.^10^ Electrodes for perforated-patch clamp recordings were prepared from thin-walled glass pipettes using a model P-87 micropipette puller (Sutter Instrument Co., CA). The electrode was filled with a KCl-based internal solution containing 150 mM KCl, and 10 mM HEPES (pH 7.2, adjusted with potassium hydroxide, 280-290 mOsm/kg). Gramicidin was dissolved in DMSO at 40 mg/ml, and then added to the pipette solution to a final concentration of 80 mg/ml. Electrodes were back filled with gramicidin-containing solution, then tip filled with gramicidin-free solution. The resistance of the patch electrode ranged from 3 to 6 MΩ. Perforated-patch recordings were made using an Axopatch 200A amplifier (Axon Instruments, CA) with a sampling frequency of 10 kHz after low-pass filtering at 1 kHz, using pClamp9 software (Clampex, Molecular Devices, CA). After the cell-attached formation, series resistance was monitored with 5 mV hyperpolarizing pulses. It typically took about 30 min for the series resistance to drop to 30-70 MΩ and stabilize. GABA (100 μM) was dissolved in recording ACSF and briefly (100-200 ms) puffed onto the dendrites of the recorded neurons via a patch-type electrode using a valve-controlled pressure application system (Picospritzer II; Parker Hannifin Corporation, OH). We allowed at least 25 s between GABA applications. The GABA_A_ receptor–mediated currents were pharmacologically isolated by application of 10 μM NBQX, and 100 μM DL-AP5 to block AMPA and NMDA receptors. The reversal potentials for the GABA-induced currents (E_GABA_) were determined by varying the holding potential of the recorded neuron in 10 mV increments, and then measuring the peak amplitude of the GABA-activated currents. Phenobarbital (100 μM) and bumetanide (10 μM) were applied alone and in combination to test their effects on GABA reversal potentials. Data were collected 10 min after drugs were applied, and each drug was applied for a total of 15 to 20 min. Recordings for each condition were compared to control recordings obtained prior to drug application. Current-voltage curves and data analysis were performed using Clampfit 9 (Molecular Devices).

**References**

1. Rakhade SN, Klein PM, Huynh T et al. Development of later life spontaneous seizures in a rodent model of hypoxia-induced neonatal seizures. Epilepsia. 2011;52:753-765

2. Sherwood NM, Timiras PS. A Stereotaxic Atlas of the Developing Rat Brain. Univ. of Calif. Press: Berkeley., 1970

3. Li Y, Cleary R, Kellogg M et al. Sensitive isotope dilution liquid chromatography/tandem mass spectrometry method for quantitative analysis of bumetanide in serum and brain tissue. J Chromatogr B Analyt Technol Biomed Life Sci. 2011;879:998-1002

4. Talos DM, Fishman RE, Park H et al. Developmental regulation of alpha-amino-3-hydroxy-5-methyl-4-isoxazole-propionic acid receptor subunit expression in forebrain and relationship to regional susceptibility to hypoxic/ischemic injury. I. Rodent cerebral white matter and cortex. J Comp Neurol. 2006;497:42-60

5. Jensen FE, Wang C, Stafstrom CE et al. Acute and chronic increases in excitability in rat hippocampal slices after perinatal hypoxia in vivo. J.Neurophysiol. 1998;79:73-81

6. Rakhade SN, Zhou C, Aujla PK et al. Early alterations of AMPA receptors mediate synaptic potentiation induced by neonatal seizures. J Neurosci. 2008;28:7979-7990

7. Pasternack M, Voipio J, Kaila K. Intracellular carbonic anhydrase activity and its role in GABA-induced acidosis in isolated rat hippocampal pyramidal neurones. Acta Physiol Scand. 1993;148:229-231

8. Kaila K, Voipio J. Postsynaptic fall in intracellular pH induced by GABA-activated bicarbonate conductance. Nature. 1987;330:163-165

9. Farrant M, Kaila K. The cellular, molecular and ionic basis of GABA(A) receptor signalling. Prog Brain Res. 2007;160:59-87

10. Talos DM, Sun H, Kosaras B et al. Altered inhibition in tuberous sclerosis and type IIb cortical dysplasia. Ann Neurol. 2012;71:539-551
